# Genetic and clinical spectrums in Korean Charcot‐Marie‐Tooth disease patients with myelin protein zero mutations

**DOI:** 10.1002/mgg3.1678

**Published:** 2021-04-06

**Authors:** Hye Jin Kim, Soo Hyun Nam, Hye Mi Kwon, Si On Lim, Jae Hong Park, Hyun Su Kim, Sang Beom Kim, Kyung Suk Lee, Ji Eun Lee, Byung‐Ok Choi, Ki Wha Chung

**Affiliations:** ^1^ Department of Health Sciences and Technology SAIHST Sungkyunkwan University Seoul Korea; ^2^ Department of Neurology Samsung Medical Center Sungkyunkwan University School of Medicine Seoul Korea; ^3^ Department of Biological Sciences Kongju National University Gongju Korea; ^4^ Department of Radiology Samsung Medical Center Sungkyunkwan University School of Medicine Seou Korea; ^5^ Department of Neurology Kyung Hee University Hospital at Gangdong Kyung Hee University School of Medicine Seoul Korea; ^6^ Department of Physics Education Kongju National University Gongju Korea; ^7^ Stem Cell & Regenerative Medicine Institute Samsung Medical Center Seoul Korea

**Keywords:** Charcot‐Marie‐Tooth disease, Korea, *MPZ*, phenotypic heterogeneity

## Abstract

**Background:**

Charcot‐Marie‐Tooth disease (CMT) is the most common disorder of inherited peripheral neuropathies characterized by distal muscle weakness and sensory loss. CMT is usually classified into three types, demyelinating, axonal, and intermediate neuropathies. Mutations in myelin protein zero (*MPZ*) gene which encodes a transmembrane protein of the Schwann cells as a major component of peripheral myelin have been reported to cause various type of CMT.

**Methods:**

This study screened *MPZ* mutations in Korean CMT patients (1,121 families) by whole exome sequencing and targeted sequencing.

**Results:**

We identified 22 pathogenic or likely pathogenic *MPZ* mutations in 36 families as the underlying cause of the CMT1B, CMTDID, or CMT2I subtypes. Among them, five mutations were novel. The frequency of CMT patients with the *MPZ* mutations was similar or slightly lower compared to other ethnic groups.

**Conclusions:**

We showed that the median onset ages and clinical phenotypes varied by subtypes: the most severe in the CMT1B group, and the mildest in the CMT2I group. This study also observed a clear correlation that earlier onsets cause more severe symptoms. We believe that this study will provide useful reference data for genetic and clinical information on CMT patients with *MPZ* mutations in Korea.

## Graphical abstract

- Mutations in myelin protein zero (MPZ) gene which encodes a transmembrane protein of the Schwann cells have been reported to cause various type of CMT.
- We identified 22 pathogenic or likely pathogenic MPZ mutations in 36 families as the underlying cause of the CMT1B, CMTDID, or CMT2I subtypes.
- The mean onset ages and clinical phenotypes varied by subtypes: the most severe in the CMT1B group, and the mildest in the CMT2I group.

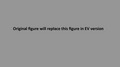

## 1 INTRODUCTION

Charcot‐Marie‐Tooth disease (CMT), also called hereditary motor and sensory neuropathy (HMSN), is a genetically and clinically heterogeneous group of progressive peripheral neuropathies characterized by distal muscle atrophy, weakness, and sensory loss. Through advances in next generation sequencing technology including whole exome sequencing and targeted gene panel sequencing, more than 130 genes have been reported to be implicated in CMT and other related disorders (Cortese et al., 2020; Gonzaga‐Jauregui et al., 2015). CMT is commonly classified into three types: demyelinating type (called CMT1) with a reduced median motor nerve conduction velocity (MNCV) of <38 m/s), axonal neuropathy (called CMT2) with a preserved or slightly reduced MNCV of >38 m/s, and intermediate type neuropathy with a MNCV of 25–45 m/s (Saporta et al., 2011). It is known that CMT exhibits a loose genotype‐phenotype correlation.

Mutations in the myelin protein zero (*MPZ*; MIM 159440) gene are implicated in above mentioned three different types of dominant neuropathies. The *MPZ* mutations have been particularly reported to cause the following subtypes: CMT1B (MIM 118200), CMT2I (MIM 607677), and CMTDID (MIM 607791) (De Jonghe et al., 1999; Hayasaka et al., 1993; Kochański, 2004; Mastaglia et al., 1999; Sanmaneechai et al., 2015; Senderek et al., 2000). Demyelinating CMT1B patients have been generally characterized by early‐onset, while axonal CMT2I patients exhibited late‐onset (Hattori et al., 2003; Shy et al., 2004). Patients with the *MPZ* mutation have shown a spectrum of different phenotypes, sometimes with phenotypic variations in the same mutation (Kochański, 2004; Mazzeo et al., 2008; Senderek et al., 2001). Mazzeo et al. (2008) reported marked phenotypic variation of the p.S78L mutation in five Italian families. Even some patients with the *MPZ* mutations have shown an intrafamilial clinical heterogeneity of mild to moderate symptoms (Senderek et al., 2001).

*MPZ*, which is strictly expressed in myelinated Schwann cells, encodes a transmembrane protein as a major component of peripheral myelin (Lemke & Axel, 1985). MPZ protein has an important role in cell adhesion and holding multiple layers of myelin sheets together tightly (Wong & Filbin, 1994; Xu et al., 2001). Several studies have reported that abnormal MPZ proteins are retained in the endoplasmic reticulum (ER) instead of being transported to the cell membrane or myelin sheath (Bai et al., 2018; Chang et al., 2019; Saporta et al., 2012; Scapin et al., 2020). Altered Mpz proteins were retained in the ER and arrested Schwann cell development in the CMT1B mouse models (Saporta et al., 2012; Scapin et al., 2020). Transgenic mice with extra copies of *Mpz* exhibited congenital demyelinating neuropathy in a dose‐dependent manner (Wrabetz et al., 2000).

*MPZ* mutations have shown wide phenotypic variation in many studied populations. We identified 22 pathogenic or likely pathogenic *MPZ* mutations in 36 families from the Korean CMT cohort study. This study grouped the patients with the *MPZ* mutations according to phenotypes and then compared the clinical characteristics among the subtypes.

## 2 PATIENTS AND METHODS

### 2.1 Ethical compliance

This study was approved by the Institutional Review Boards of Sungkyunkwan University, Samsung Medical Center (2014‐08‐057‐002), and Kongju National University (KNU‐IRB‐2018‐62). Written informed consent was obtained from all the participants.

### 2.2 Patients

This study was conducted with a cohort of 1,121 unrelated Korean CMT families. From the analysis of the copy number variation in the 17p12 region, 353 families were determined to be CMT1A (MIM 118220) with *PMP22* (MIM 601097) duplication, and the remaining 768 families were further investigated to find the *MPZ* mutations.

### 2.3 Clinical and electrophysiological examinations

Clinical and electrophysiological examinations were performed by the methods of Lee, Nam, Choi, Noh, et al. (2020). The strengths of the flexor and extensor muscles were measured using the standard Medical Research Council (MRC) scale. Physical disability condition was determined by the CMT neuropathy score (CMTNS) version 2 and the functional disability scale (FDS). Patients were divided into three categories: mild (CMTNS ≤10), moderate (CMTNS 11–20), and severe (CMTNS ≥21) according to the CMTNS values.

Motor and sensory NCVs were determined by surface stimulation and recording electrodes. MNCVs and compound muscle action potentials (CMAPs) of the median and ulnar nerves were measured by stimulating the elbow and wrist and the abductor pollicis brevis and adductor digiti quinti, respectively. The MNCVs and CMAPs of the peroneal and tibial nerves were measured by stimulating the knee and ankle and the extensor digitorum brevis and adductor hallucis, respectively. CMAP amplitudes were measured from the baseline to the negative peak values. Sensory nerve action potentials (SNAPs) were measured from the positive peaks to the negative peaks. Sensory nerve conduction velocities (SNCVs) were determined over a finger‐wrist segment from the median and ulnar nerves by orthodromic scoring.

### 2.4 Lower extremity MRI

MRIs of the lower extremities (pelvic girdle, bilateral thigh, and lower leg) were obtained using either a 1.5‐T or 3.0‐T MRI system (Siemens Healthcare, Frankfurt, Germany). MRIs were bilaterally obtained from three levels (proximal, mid, and distal) of the thigh muscles and two levels (proximal and distal) of the lower leg muscles. The axial T1‐weighted turbo spin‐echo images of the thigh and lower leg muscles were graded into a five‐point semiquantitative scale by the degree of fatty infiltration (Goutallier et al., 1994).

### 2.5 Genetic study

Genomic DNA was extracted from whole blood samples using the QIAamp DNA Mini Kit (Qiagen, Hilden, Germany). For the patients who were negative for 17p12 (*PMP22*) duplication and deletion, the *MPZ* mutations were screened using whole exome sequencing (WES) or targeted sequencing of inherited peripheral neuropathy genes (Lee, Nam, Choi, Nam, et al., 2020). The exome was captured using the SeqCap EZ v2.0 (Roche‐NimbleGen, Madison, WI, USA) or the SureSelect Human All Exon 50 M Kit (Agilent Technologies, Santa Clara, CA, USA), and sequencing was performed by the HiSeq2000 or HiSeq2500 Genome Analyzer (Illumina, San Diego, CA, USA).

The human genome UCSC assembly hg19 was used as the reference sequence for mapping and annotation (http://genome.ucsc.edu). From the called variant data, functionally significant variants (missense, nonsense, exonic indel and splicing site variants) were first selected, and then, unreported or rare variants with minor allele frequencies of ≤0.01 were further chosen in the dbSNP154 (http://www.ncbi.nlm.nih.gov), the 1000 Genomes project database (http://www.1000genomes.org/), the Exome Variant Server (EVS, http://evs.gs.washington.edu/EVS/), the Genome Aggregation Database (gnomAD) v2.1.1 (https://gnomad.broadinstitute.org/), and the Korean Reference Genome Database (KRGDB, http://coda.nih.go.kr/coda/KRGDB/). Pathogenic candidates of the variants were confirmed by Sanger sequencing using the genetic analyzers ABI3130XL or SeqStudio (Life Technologies‐Thermo Fisher Scientific, Foster City, CA, USA).

### 2.6 Conservation, in silico prediction, and determination of pathogenicity

Conservation analysis of the protein sequences was performed using MEGA6, ver. 6.0 (http://www.megasoftware.net/). The putative conformational changes that could be induced by the mutations were evaluated by inspecting the crystal structure of the human MPZ (Protein Data Bank, PDB ID: 3OAI, http://www.rscb.org/) (Liu et al., 2012). In silico prediction of the pathogenicity of the variants was performed by the algorithms of PROVEAN (http://provean.jcvi.org/seq_submit.php), PolyPhen‐2 (http://genetics.bwh.harvard.edu/pph2/), MUpro (http://www.ics.uci.edu/~baldig/mutation), and Fathmm (http://fathmm.biocompute.org.uk/). The pathogenicity was basically determined according to the American College of Medical Genetics and Genomics (ACMG) guideline (Richards et al., 2015).

### 2.7 Statistical analysis

All values are expressed as the median (interquartile range) or mean ± standard deviation (SD) in clinical and electrophysiological features respectively. The pairwise comparisons among the subgroups were evaluated by Student's *t*‐test and one‐way analysis of variance. Correlations were determined using Pearson's correlation coefficient (*r*). The statistical significance was determined at the level of *p* < 0.05.

## 3 RESULTS

### 3.1 Identification of *MPZ* mutations in Korean CMT patients

WES or targeted sequencing using CMT or the CMT‐related gene panel was applied to the probands of 768 CMT families who were negative for *PMP22* duplication. From the genomic screening, 22 pathogenic or likely pathogenic *MPZ* mutations were identified in 36 unrelated families with 60 patients (Table 1). Of the mutations, 11 mutations (p.C50Y, p.S78L, p.H81Q, p.H81R, p.R98C, p.R98L, p.C127S, p.P132A, p.G137D, p.L175 fs*74, and p.X249E*64) have been previously reported to cause CMT1B (Choi et al., 2011; DiVincenzo et al., 2014; Farwell et al., 2015; Milovidova et al., 2011; Nelis et al., 1994; Østern et al., 2013; Rouger et al., 1996; Sorour et al., 1997; Wang et al., 2016; Warner et al., 1996; Xu et al., 2019), and four mutations (p.Y88H, p.Q100X, p.T124M, and p.K236E) have been reported to cause CMT2I (Choi et al., 2004; Miltenberger‐Miltenyi et al., 2009; Nam et al., 2016; Schiavon et al., 1998). The c.449‐1G>T splicing acceptor site mutation was particularly identified in five families consisting of one CMT1B and four CMTDID families (Choi et al., 2004). The p.F52V was also found in both patient types of CMT1B and CMT2I (Nam et al., 2016).

**TABLE 1:** *MPZ* mutations in the Korean CMT patients

Nucleotide changes	Amino acid changes	No. of families (Family ID)	CMT type	References
c.149G>A	p.C50Y	1 (FC619)	CMT1B	Milovidova et al. (2011)
c.154T>G	p.F52V	2 (FC156, FC157)	CMT1B, CMT2I	Nam et al. (2016)
c.155T>G	p.F52C	1 (FC611)	CMT1B	This study
c.233C>T	p.S78L	3 (FC202, FC626, FC1200)	CMT1B	Nelis et al. (1994)
c.242A>G	p.H81R	1 (FC1159)	CMT1B	Sorour et al. (1997)
c.243C>G	p.H81Q	2 (FC133, FC1072)	CMT1B	Choi et al. (2011)
c.262T>C	p.Y88H	1 (FC141)	CMT2I	Nam et al. (2016)
c.292C>T	p.R98C	3 (FC263, FC508, FC533)	CMT1B	Rouger et al. (1996)
c.293G>T	p.R98L	1 (FC987)	CMT1B	Wang et al. (2016)
c.298C>T	p.Q100X	2 (FC486, FC930)	CMT2I	Miltenberger‐Miltenyi et al. (2009)
c.358_360del	p.S120del	1 (FC304)	CMT1B	This study
c.371C>T	p.T124M	1 (FC658)	CMT2I	Schiavon et al. (1998)
c.380G>C	p.C127S	1 (FC560)	CMT1B	Farwell et al. (2015)
c.394C>G	p.P132A	1 (FC452)	CMT1B	Xu et al. (2019)
c.394C>T	p.P132S	2 (FC203, FC572)	CMT1B	This study
c.398C>T	p.P133L	1 (FC201)	CMT1B	This study
c.410G>A	p.G137D	1 (FC240)	CMT1B	Østern et al. (2013)
c.449‐1G>T	Splicing acceptor site	5 (FC019, FC164, FC509, FC797, FC943)	CMT1B, CMTDID	Choi et al. (2004)
c.522_525del	p.L175fs*74	1 (FC455)	CMT1B	Warner et al. (1996)
c.659A>G	p.Y220C	2 (FC336, FC596)	CMT1B	This study
c.706A>G	p.K236E	2 (FC015, FC559)	CMT2I	Choi et al. (2004)
c.745T>G	p.X249E*64	1 (FC1027)	CMT1B	DiVincenzo et al. (2014)

Abbreviation: CMT, Charcot‐Marie‐Tooth disease.

**FIGURE 1:**
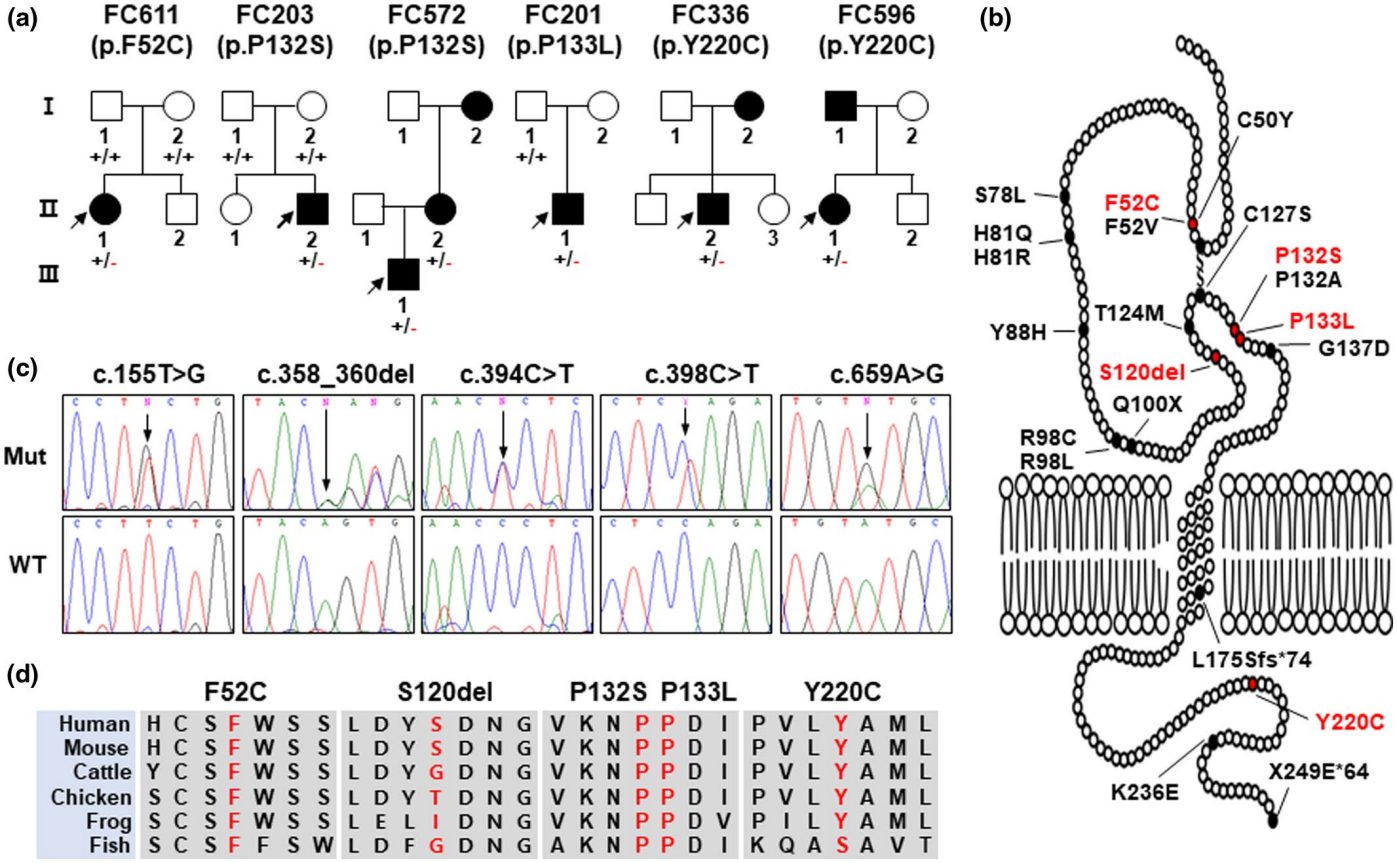
Novel *MPZ* mutations in the CMT families. (a) The pedigrees and genotypes of 6 CMT families with novel *MPZ* mutations (□, ○: unaffected members; ■, ●: affected members). (b) Schematic structure of the MPZ protein and location of the identified mutations in this study. Mutations indicated by red color are unreported mutations. (c) Sequencing chromatograms of the novel *MPZ* mutations. Vertical arrows indicate the mutation sites (Mut: mutant allele, WT: wild‐type allele). (d) Conservation analysis of the amino acid sequences on the mutation sites (Human: NP_000521.2, mouse: NP_001302428.1, cattle: NP_001072975.1, chicken: NP_001345858.1, frog: NP_001072741.1, and zebrafish: XP_017213076.1)

Five mutations (p.F52C, p.S120del, p.P132S, p.P133L, and p.Y220C) from seven families were determined to be unreported novel mutations (Table 2, Figure 1a,b). At the same amino acid residues of the p.F52C and p.P132S mutations, p.F52V and p.P132A were previously reported as the pathogenic mutations, respectively. All the novel mutations were confirmed by Sanger sequencing (Figure 1c). They were not reported in the 1000 Genomes Project, EVS, gnomAD, and KRGDB databases, except for registration of p.Y220C with a very low minor allele frequency (4.0E‐06) in the gnomAD. Most mutation sites were highly conserved in different vertebrate species (Figure 1d), and all these mutations were predicted to affect protein function by several in silico analyses (PROVEAN: −9.492–3.141; PolyPhen‐2: 0.988–1.000; and MUpro: −0.998–0.552; Fathmm: −3.54–0.00) (Table 2).

**TABLE 2:** Five novel *MPZ* mutations found in this study

Mutation		No. of families	Type	dbSNP accession No	Mutant allele frequencies				In silico analysis				ACMG
Nucleotide	Amino acid				1000G	gnomAD	EVS	KRGDB	PROVEAN	Polyphen‐2	MUpro	Fahmm	
c.155T>G	p.F52C	1	CMT1B	–	–	–	–	–	−7.532^a^	1.000^a^	−0.916^a^	−3.54^a^	LP
c.358_360del	p.S120del	1	CMT1B	–	–	–	–	–	−9.492^a^	–	–	–	LP
c.394C>T	p.P132S	2	CMT1B	rs1553259649	–	–	–	–	−6.841^a^	0.988^a^	−0.998^a^	0.00	LP
c.398C>T	p.P133L	1	CMT1B	rs1558154010	–	–	–	–	−9.044^a^	0.997^a^	−0.878^a^	−3.30^a^	LP
c.659A>G	p.Y220C	2	CMT1B	rs767339597	–	4.0E‐06	–	–	−3.141^a^	0.999^a^	0.552	−3.14^a^	LP

Abbreviations: 1000G, 1000 Genomes database; ACMG, American College of Medical Genetics and Genomics guideline; CMT1B, Charcot‐Marie‐Tooth disease type 1B; EVS, Exome Variant Server; gnomAD, Genome Aggregation Database; KRGDB, Korean Reference Genome Database; LP, likely pathogenic.

^a^ Pathogenic prediction.

**FIGURE 2:**
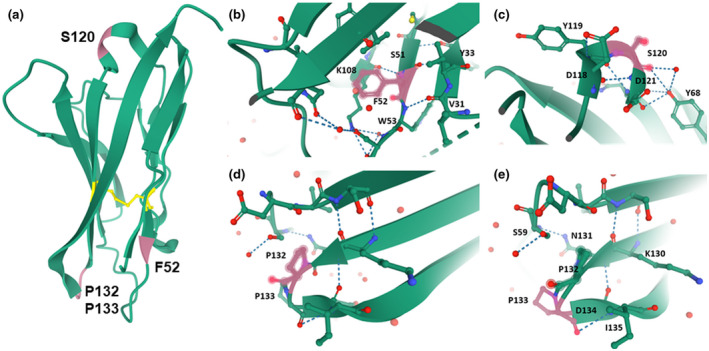
3D structure of the extra‐cellular domain of the MPZ protein and the sites of the novel mutations. (a) The crystal structure of the extra‐cellular domain is depicted in green. The location of four of the five novel mutations, F52, S120, P132, and P133 are colored in pink, while the intra‐molecular disulfide bond between C50 and C127 is shown in yellow. (b‐e) A zoom‐in view of the mutated residues and their surroundings. (b) The benzene ring of F52 (pink) is located in between two β strands. (c) The S120 (pink) is located in a helix forming hydrogen bonds (dashed) to the surroundings. (d, e) The P132 and P133 residues (pink) are located in a proline‐hinge connecting two β strands. These 3D structures were visualized using the Mol* feature of Protein Data Bank (http://www.rcsb.org)

Four of the five novel mutations are located at the extracellular domain of MPZ, and their crystal structures have been resolved (Liu et al., 2012). MPZ is expected to function as a homotetramer (Shapiro et al., 1996), and several clinically important mutations (p.R98C, p.R98H, p.T114M, and p.H81R) are putatively associated with the disruption of the tetramer assembly (Liu et al., 2012). According to the protein structure, these novel mutants might influence inter‐ and intra‐tetramer interactions. According to the homotetramer model, F52 and S120 are located at the intermolecular interfaces; therefore, the mutations at these positions are presumed to weaken the tetramer assembly. The location of the mutation p.F52C is not desirable either because it adds another cysteine residue in the vicinity of the intramolecular disulfide bond (C50‐C127, Figure 2a) that has a crucial role in the stability of the protein. Furthermore, the removal of the benzene ring that stabilizes the interaction between two β strands will be unfavorable (Figure 2b). As for mutation p.S120del, 9 hydrogen bonds will be lost with that deletion (Figure 2c). Finally, mutations p.P132S and p.P133L are located at the proline‐hinge; therefore, these mutations will alter the structure of the protein (Figure 2d,e).

Of the 20 trio families with father‐mother‐sibling(s), de novo mutations were observed in 9 families (p.C50Y in FC619, p.F52C in FC611, p.H81Q in FC1072, p.R98C in FC508, p.R98L in FC987, p.P132S in FC203 and FC572, p.L175del in FC455, and p.X249E*64 in FC1027) at a rate of 0.45. The frequency of CMT patients with the *MPZ* mutations was determined to be 3.2% in the total independent patients and 4.7% in the patients negative for *PMP22* duplication (Table 3). These frequencies were similar with those of the China (3.3% and 6.4%) (Hsu et al., 2019) and Britain (3.1% and 5.1%) (Murphy et al., 2012), but were relatively lower than those of most other examined countries.

**TABLE 3:** Frequencies of CMT patients with *MPZ* mutations in various populations based on a literature review

Populations	Frequencies		References
	Total CMT patients (%)	CMT patients excluding CMT1A (%)	
Korean	3.2	4.7	This study
Chinese	3.3	6.4	Hsu et al. (2019)
Japanese	5.1	NA	Yoshimura et al. (2019)
German	4.2	6.4	Gess et al. (2013)
British	3.1	5.1	Murphy et al. (2012)
American	4.1	6.5	Fridman et al. (2015)
Spanish	4.3	7.5	Sivera et al. (2013)
Italian	4.3	12.3	Manganelli et al. (2014)
Hungarian	4.5	7.5	Milley et al. (2018)
Norwegian	6.0	NA	Østern et al. (2013)
Russian	3.5	5.2	Mersiyanova et al. (2000)
Finnish	5.2	NA	Silander et al. (1998)
Austrian	4.0	NA	Miltenberger‐Miltenyi et al. (2009)

Abbreviations: CMT, Charcot‐Marie‐Tooth disease 1A; NA, not available.

### 3.2 Earlier onset and severe disability in CMT1B subtype

When the clinical features of the 60 patients with *MPZ* mutations were analyzed, they were classified into 48 CMT1B (22 males and 26 females), 5 CMTDID (4 males and 1 female), and 7 CMT2I (6 males and 1 female) (Table 4). The median onset ages were 5.0 (2.0–12.0) years for CMT1B, 15.0 (15.0–20.0) years for CMTDID, and 36.0 (29.5–51.0) years for CMT2I. The pairwise comparisons showed significant differences between CMT1B and CMTDID (*p* = 0.025) and between CMT1B and CMT2I (*p* < 0.001), but not significant between CMTDID and CMT2I (*p* = 0. 053).

**TABLE 4:** Clinical and electrophysiological features in CMT patients with *MPZ* mutations

Items	CMT1B	CMTDID	CMT2I	*p*‐value			
				1B vs. DID	1B vs. 2I	DID vs. 2I	ANOVA
Patient number	48	5	7				
Female ratio	54%	20%	14%	0.490	0.263	0.793	.
Examined age (years)	21.0 (13.0–40.0)	33.0 (25.0–50.0)	51.0 (44.0–57.0)	0.129	0.002	0.332	0.005
Onset age (years)	5.0 (2.0–12.0)	15.0 (15.0–20.0)	36.0 (29.5–51.0)	0.025	>0.001	0.053	>0.001
Disability score							
CMTNS	15.0 (13.0–22.0)	9.5 (9.0–10.3)	7.0 (6.0–9.0)	0.102	0.004	0.326	0.023
FDS	2.5 (2.0–3.0)	2.0 (2.0–2.0)	1.0 (1.0–1.5)	0.092	0.022	0.092	0.004
Nerve conduction studies							
Patient number	38	4	5				
Median motor nerve							
CMAP (mV)	6.0 ± 5.6	12.8 ± 1.9	13.0 ± 4.3	0.033	0.011	0.915	0.006
MNCV (m/s)	12.2 ± 11.0	41.3 ± 3.1	46.0 ± 6.6	>0.001	>0.001	0.431	>0.001
Peroneal nerve							
CMAP (mV)	0.9 ± 2.0	4.0 ± 3.1	2.1 ± 2.8	0.001	0.231	0.132	0.002
MNCV (m/s)	5.1 ± 9.0	25.7 ± 17.2	20.4 ± 19.4	>0.001	0.004	0.278	>0.001
Median sensory nerve							
SNAP (μV)	2.3 ± 4.7	10.8 ± 15.3	15.6 ± 12.5	>0.001	>0.001	0.718	>0.001
SNCV (m/s)	7.4 ± 11.7	18.0 ± 25.5	34.4 ± 4.3	0.021	>0.001	0.780	>0.001
Sural nerve							
SNAP (μV)	1.1 ± 3.5	6.9 ± 9.7	5.4 ± 5.9	0.001	0.059	0.346	0.002
SNCV (m/s)	3.4 ± 9.0	13.3 ± 18.7	17.2 ± 14.9	0.016	0.020	0.643	0.007

Note

Normal NCV values: motor median nerve ≥50.5 m/s; sensory median nerve ≥39.3 m/s; sural nerve ≥32.1 m/s. Normal amplitude values: motor median nerve ≥6 mV; sensory median nerve ≥8.8 μV; sural nerve ≥6.0 μV.

Abbreviations: ANOVA, analysis of variance; FDS, functional disability scale; CMTNS, Charcot‐Marie‐Tooth neuropathy score; CMAP, compound muscle action potential; CMT, Charcot‐Marie‐Tooth disease; MNCV, motor nerve conduction velocity; SNAP, sensory nerve action potential; SNCV, sensory nerve conduction velocity.

**FIGURE 3:**
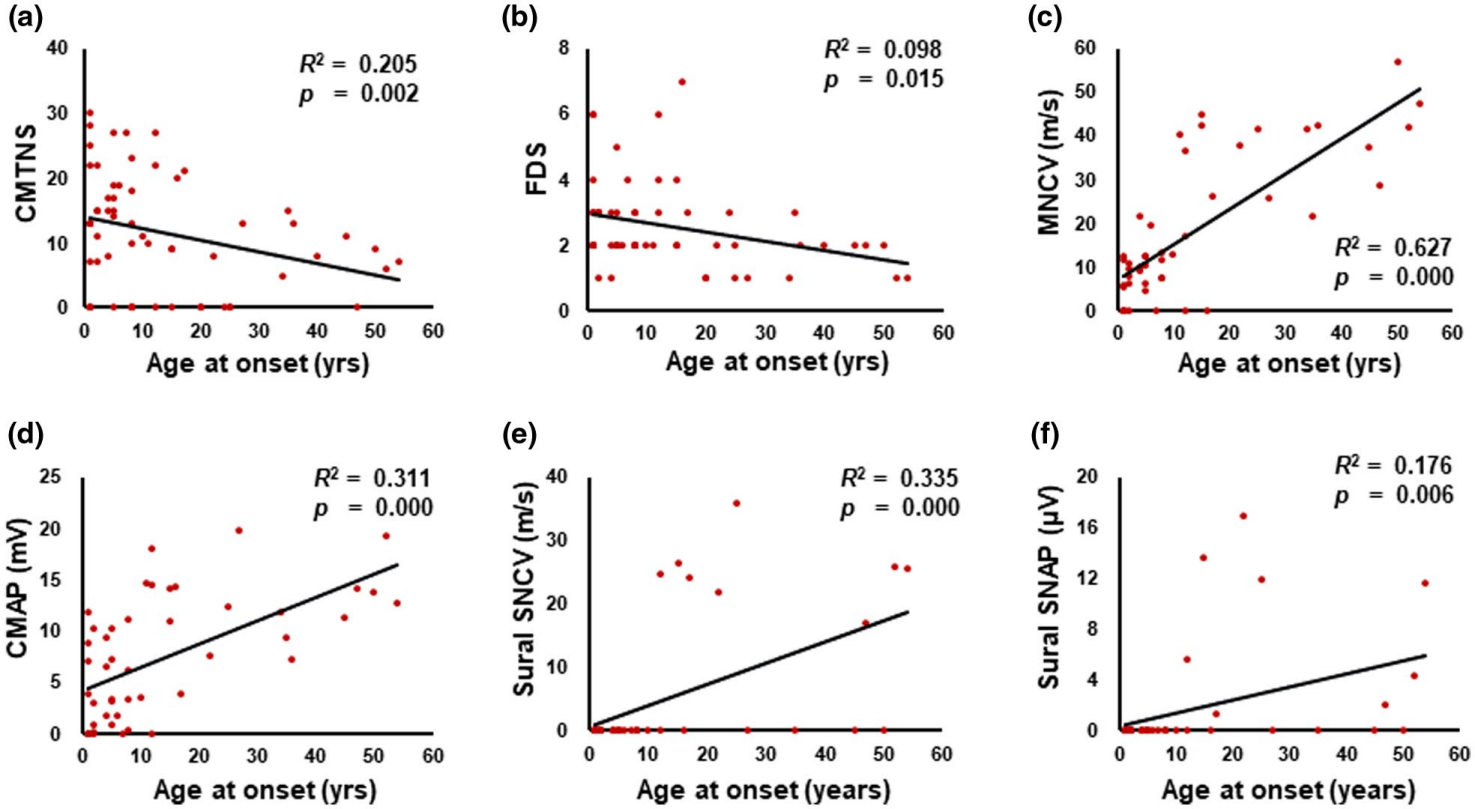
Scatter plot diagrams with Pearson's correlation analysis between the onset ages and clinical phenotypes. (a) Onset vs. CMT neuropathy score (CMTNS), (b) Onset vs. functional disability scale (FDS). (c) Onset vs. median motor nerve conduction velocity (MNCV), (d) Onset vs. median motor nerve compound muscle action potential (CMAP), (e) Onset vs. sural sensory nerve conduction velocity (SNCV), and (f) Onset vs. sural sensory nerve action potential (SNAP)

When the disease disabilities were determined by the measurement of CMTNS and FDS, the CMT1B group showed the most severe symptom, while CMT2I showed the mildest symptom with significant differences (CMTNS: *p* = 0.004, and FDS: *p* = 0.022). Although the CMTNS and FDS values of the CMT1B group were higher than those of the CMTDID group, no significant differences were found. In the CMT1B group, based on the CMTNS, patients with moderate disabilities were the most common (53%) and then severe patients (31%) and mild patients (17%). In the CMTDID and CMT2I groups, mild patients were the most common with frequencies of 75% and 80%, respectively. When the correlation between onset ages and clinical disability were examined, earlier onset was significantly correlated with severe disability in both comparisons of onset vs. CMTNS (*p* = 0.002, Figure 3a) and onset vs. FDS (*p* = 0.015, Figure 3b).

### 3.3 More decreased NCVs and action potentials in CMT1B

Motor and sensory nerve conduction studies were performed on the 48 CMT patients with *MPZ* mutations (Table 4). The mean median MNCV of the CMT1B patients was 12.2 ± 11.0 m/s, which was significantly lower than those of CMTDID (41.3 ± 3.1 m/s, *p* < 0.001) and CMT2I (46.0 ± 6.6 m/s, *p* < 0.001). The mean median SNCV (7.4 ± 11.7 m/s) of the CMT1B patients was also significantly lower than those of CMTDID (18.0 ± 25.5 m/s, *p* = 0.021) and CMT2I (34.4 ± 4.3 m/s, *p* < 0.001). Moreover, the peroneal MNCV and sural SNCV were significantly decreased in the CMT1B patients compared to those in the CMTDID or CMT2I patients. The median motor nerve CMAP (6.0 ± 5.6 mV) of the CMT1B group was largely decreased compared to those of the CMTDID (12.8 ± 1.9 mV, *p* = 0.033) and CMT2I patients (13.0 ± 4.3 mV, *p* = 0.011). The CMAP of the peroneal nerve and the SNAPs of the median and sural nerves were also significantly decreased in the CMT1B patients than those in the CMTDID and CMT2I patients. However, no significant difference was observed in the nerve conduction velocities and action potentials between CMTDID and CMT2I.

The analysis of the Pearson's correlation between onset ages and electrophysiological values showed that earlier onset was significantly correlated with more severely decreased NCVs and action potentials, i.e., onset vs. median MNCV (*p* < 0.001, Figure 3c), onset vs. median CMAP (*p* < 0.001, Figure 3d), onset vs. sural SNCV (*p* < 0.001, Figure 3e), and onset vs. sural SNAP (*p* = 0.006, Figure 3f). These results are consistent with the result that when the onset age is earlier, the clinical disability is more severe.

### 3.4 Lower limb MRI features

The thigh and calf MRIs were obtained from 17 patients, who were consisted of 15 CMT1B, one CMTDID, and one CMT2I (Table S1 and Table S2). Follow‐up MRIs were obtained in three patients (FC619‐1, FC452‐1, and FC455‐1).

**FIGURE 4:**
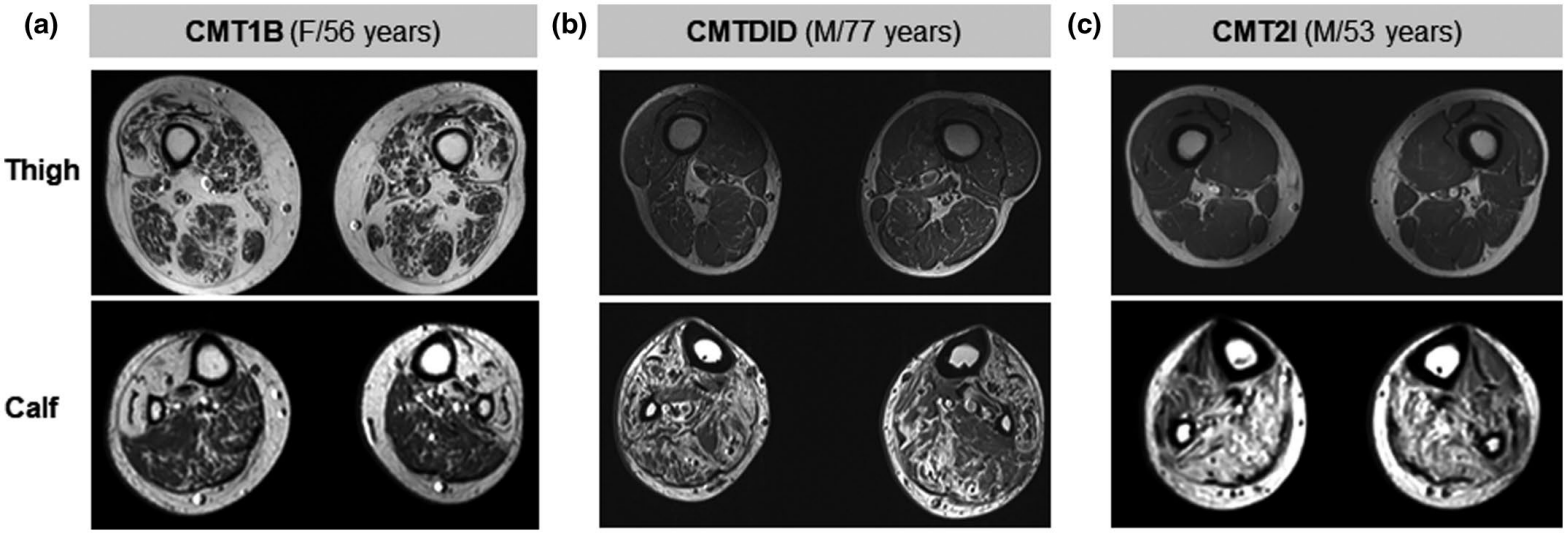
Axial T1‐weighted MRIs of the thigh (up) and calf (down) leg in the CMT1B, CMTDID, and CMT2I patients. (a) A 56‐year‐old female CMT1B patient (FC1159: p.H81R) showing total fat replacement of the anterior and lateral compartment muscles in the distal lower leg and severe (grades 3 and 4) fat infiltration in anterior and posterior compartment muscles in the distal thigh. (b) A 77‐year‐old male CMTDID patient (FC943: c.449‐1G>T) showing severe (grades 3 and 4) fat infiltration in nearly all compartment muscles of the distal lower leg and mild fat infiltration in the distal thigh muscles. (CF) A 53‐year‐old male CMT2I patient (FC658: p.T124M) showing severe fat infiltration in the anterior, superficial, and deep posterior compartment muscles of the distal lower leg and mild fat infiltration in the thigh muscles

CMT1B patients with MRI consisted of five males and ten females who were mostly in their first to third decades of life. Most patients demonstrated mild (grade 1 and 2) fat infiltrations in the calf leg and thigh muscles. However, among the impaired muscles, the anterior, lateral, and superficial posterior compartment muscles of the distal lower legs showed severe (grades 3 and 4) fat infiltrations in some patients. A 56 year‐old female patient (FC1159) with p.H81R mutation showed almost total fat replacement of anterior and lateral compartment muscles of the lower leg along their entire length and anterior and posterior compartment muscles in the distal thigh also showed severe fat infiltration (Figure 4a). In a CMTDID patient who was a 77‐year‐old male (FC943) with the c.449‐1G>T mutation, he had severe (grade 3 and 4) fat infiltration in nearly all compartment muscles of the distal lower leg and in the superficial posterior compartment muscles of the proximal lower leg (Figure 4b). However, the thigh muscles showed a relatively mild fat infiltration. In a CMT2I patient, a 53‐year‐old male (FC658) with the p.T124M mutation showed severe (grades 3 and 4) fat infiltration in the anterior, superficial, and deep posterior compartment muscles of the distal lower leg, while mild fat infiltrations were seen in the thigh muscles (Figure 4c).

## 4 DISCUSSION

From the Korean CMT cohort, this study identified 22 pathogenic or likely pathogenic *MPZ* mutations in 36 families consisting of three different CMT subtypes, CMT1B, CMTDID, and CMT2I, and then analyzed the clinical and electrophysiological differences between the patient subtypes. The c.449‐1G>T splicing site mutation was observed in five independent families, and the p.S78L and p.R98C mutations were observed in each of the three families. Since these mutations have also been reported several times in different ethnic groups (Nelis et al., 1994; Rouger et al., 1996; Song et al., 2006), these sites are suggested as mutational hot spots. The c.233C and c.292C are located at the CpG sites, which may be partly related to the frequent mutations. The c.449‐1G>T and p.F52V mutations were particularly identified in two types of affected individuals (CMT1B and CMTDID for c.449‐1G>T and CMT1B and CMT2I for p.F52V). Some previous studies have reported phenotypic heterogeneities for the same *MPZ* mutations (Mazzeo et al., 2008; Senderek et al., 2001). The rate of de novo mutations was determined to be 45.0% of the trio CMT families with the *MPZ* mutations. This rate is significantly higher compared to the de novo CMT1A cases in Korea (18.7%) (Lee, Nam, Choi, Noh, et al., 2020).

Of the 22 *MPZ* mutations, five mutations were novel: a nonframeshift deletion (p.S120del), and four were missense (p.F52C, p.P132S, p.P133L, and p.Y220C) mutations. These novel mutations were cosegregated with the affected individual(s) within each family and were not reported in the Korean genome databases (KRGDB) or in global public human genome databases (such as 1000 Genomes project, gnomAD, and EVS). The novel mutation sites are well conserved among different animal species, and several in silico analyses predicted that all the missense mutations affect the protein structure. Furthermore, inspection of the mutations in the crystal structure suggests disruption of intra‐molecular interactions and impaired MPZ tetramer assembly.

The frequency of the *MPZ* mutations was determined to be 3.2% in the total independent patients diagnosed with CMT and 4.7% in the patients negative for *PMP22* duplication. The frequencies of CMT patients with *MPZ* mutations are different depending on the ethnic groups. The frequency of CMT patients with the *MPZ* mutations from the total Korean cases was similar with Chinese (3.3%), British (3.1%), and Russian patients (3.5%) (Hsu et al., 2019; Mersiyanova et al., 2000; Murphy et al., 2012). However, the Korean frequency was somewhat lower than those in most of the other examined countries: from 4.0% in Austrians to 6.0% in Norwegians (Fridman et al., 2015; Gess et al., 2013; Manganelli et al., 2014; Milley et al., 2018; Miltenberger‐Miltenyi et al., 2009; Østern et al., 2013; Silander et al., 1998; Sivera et al., 2013; Yoshimura et al., 2019). When we compared the frequencies of patients with the *MPZ* mutations among patients excluding CMT1A, the frequency of Koreans was also lower than those of most other populations.

When disease disability was compared among different groups, the degree of severity was determined to be in the order of CMT1B, CMTDID, and CMT2I, based on the CMTNS and FDS. Similarly, the average onset ages were also earlier in the same order. In most of the nerves examined, the NCVs and action potentials were significantly decreased in the CMT1B group compared to the other two groups. Those results were similar in previous reports (Hattori et al., 2003). The CMTDID group showed slightly lower electrophysiological values compared to the CMT2I group, but there was no significant difference.

In the affected individuals with the *MPZ* mutations, significant correlations were found between the onset ages and clinical phenotypes. When the correlation between onset ages and clinical disability were examined, an earlier onset was positively correlated with worse symptoms in both CMTNS and FDS. Significant correlations were also found in motor and sensory NCVs and action potentials: when the onset was earlier, the values were more decreased.

MRI analyses revealed varying degrees of intramuscular fat infiltration in the lower extremity muscles of *MPZ* patients. CMT1B‐type patients mostly showed mild (grades 1 and 2) fat infiltration in both the thigh and lower leg. In comparison of a 56 year‐old CMT1B patient with CMTDID patient (77 year‐old) and CMT2I patient (53 year‐old), the CMT1B patient showed more severe fat infiltration involving the distal thigh muscles. While CMTDID and CMT2I patients showed only mild fat infiltration. In the lower leg, the CMT1B patient showed prominent predilection for anterior and lateral compartment muscles, demonstrating total fat replacement of the muscles in their entire length. In contrast, the CMT2I patient showed severe fat infiltrations predominantly involving superficial and deep posterior compartment muscles. The CMTDID showed severe fat infiltration in nearly all compartment muscles of the distal lower leg. These difference in MRI findings may suggest difference of primarily affected muscle group among the three groups. Yet, further study including larger number of patients with diverse age is needed to clarify this difference.

In conclusion, our cohort study identified 22 *MPZ* mutations including five novel mutations as the underlying cause of the CMT1B, CMTDID and CMT2I subtypes. It seems that the frequency of the *MPZ* mutations was similar or slightly low compared to other ethnic groups. This study found that the median onset ages and clinical phenotypes were different according to the subtypes. We also observed a clear correlation that earlier onsets cause more severe symptoms. We believe that this study will provide useful reference data for the genetic and clinical information on CMT patients with *MPZ* mutations in Korea.

## ETHICAL COMPLIANCE

This study was performed in accordance with the protocol approved by the Institutional Review Boards of Sungkyunkwan University, Samsung Medical Center (2014‐08‐057‐002), and Kongju National University (KNU‐IRB‐2018‐62). Written informed consent was obtained from all the participants.

## CONFLICT OF INTEREST

The authors declare no competing interests.

## AUTHOR CONTRIBUTIONS

Byung‐Ok Choi and Ki Wha Chung planed and supervised this study. Hye Jin Kim, Soo Hyun Nam, Hye Mi Kwon, and Si On Lim performed molecular genetic works. Soo Hyun Nam, and Jae Hong Park performed clinical works. Hye Jin Kim, Kyung Suk Lee, Ji Eun Lee, and Ki Wha Chung interpreted genetic data and statistical analysis. Sang Beom Kim, and Byung‐Ok Choi collected the participants’ samples and information. Hye Jin Kim, Byung‐Ok Choi, and Ki Wha Chung wrote the manuscript. All the co‑authors read and approved the final version of the manuscript.

## Supporting information

Table S1‐S2

## Data Availability

All raw genetic and clinical data generated or analyzed during this study are available upon request to the corresponding author.
