# Supplementary material for: Genetic and clinical spectrums in Korean Charcot‐Marie‐Tooth disease patients with myelin protein zero mutations
Source: Mol Genet Genomic Med. 2021 Apr 6;9(6):e1678. doi: 10.1002/mgg3.1678 (PMC8222852; doi:10.1002/mgg3.1678)
Supplement: Supplementary file 1 — Table S1‐S2 [file MGG3-9-e1678-s001.docx]

**Table S1.** Thigh MRI features in Charcot-Marie-Tooth patients with *MPZ* mutations

| Patient | Type | Sex | Age at MRI | Level | Anterior compartment | | | | | Medial compartment | | | | Posterior compartment | | |
| --- | --- | --- | --- | --- | --- | --- | --- | --- | --- | --- | --- | --- | --- | --- | --- | --- |
|  |  |  |  |  | Sartorius | Rectus femoris | Vastus intermedius | Vastus lateralis | Vastus medialis | Adductor longus | Adductor brevis | Adductor magnus | Gracilis | Semitendinosus | Semimembranosus | Biceps femoris |
| FC619-1 | CMT1B | M | 3 | Proximal | 0/0 | 0/0 | 0/0 | 0/0 | 0/0 | 0/0 | 0/0 | 0/0 | 0/0 | 0/0 | N/A | N/A |
|  |  |  |  | Mid | 0/0 | 0/0 | 0/0 | 0/0 | 0/0 | 0/0 | 0/0 | 0/0 | 0/0 | 0/0 | 0/0 | 0/0 |
|  |  |  |  | Distal | 0/0 | 0/0 | 0/0 | 0/0 | 1/1 | N/A | N/A | N/A | 0/0 | 0/0 | 0/0 | 0/0 |
|  |  |  | 7 | Proximal | 1/2 | 0/0 | 0/0 | 0/0 | 0/0 | 0/0 | 0/0 | 0/0 | 0/0 | 0/0 | N/A | N/A |
|  |  |  |  | Mid | 0/1 | 0/0 | 0/0 | 0/0 | 0/0 | 0/0 | 0/0 | 0/0 | 1/0 | 0/0 | 1/0 | 0/0 |
|  |  |  |  | Distal | 1/1 | 0/0 | 0/0 | 0/0 | 0/0 | N/A | N/A | N/A | 0/0 | 0/0 | 0/0 | 0/0 |
| FC611-1 | CMT1B | F | 3 | Proximal | 0/0 | 0/0 | 0/0 | 0/0 | 0/0 | 0/0 | 0/0 | 0/0 | 0/0 | 0/0 | N/A | N/A |
|  |  |  |  | Mid | 1/1 | 0/0 | 1/0 | 0/0 | 0/0 | 0/0 | 0/0 | 1/1 | 1/1 | 1/1 | 1/1 | 0/0 |
|  |  |  |  | Distal | 1/1 | 0/0 | 0/1 | 1/0 | 0/0 | N/A | N/A | N/A | 1/1 | 1/1 | 1/1 | 1/1 |
| FC133-4 | CMT1B | M | 15 | Proximal | 0/0 | 0/0 | 0/0 | 0/0 | 0/0 | 0/0 | 0/0 | 0/0 | 0/0 | 0/0 | N/A | N/A |
|  |  |  |  | Mid | 0/0 | 0/0 | 0/0 | 0/0 | 0/0 | 0/0 | 0/0 | 0/0 | 1/0 | 1/1 | 1/1 | 1/1 |
|  |  |  |  | Distal | 1/0 | 0/0 | 0/0 | 0/0 | 0/0 | N/A | N/A | N/A | 1/1 | 0/0 | 1/1 | 1/1 |
| FC133-10 | CMT1B | M | 19 | Proximal | 1/1 | 0/1 | 1/1 | 1/0 | 1/1 | 1/0 | 0/0 | 1/1 | 0/0 | 1/0 | N/A | N/A |
|  |  |  |  | Mid | 1/1 | 0/0 | 0/0 | 0/1 | 0/0 | 0/0 | 0/0 | 1/1 | 1/1 | 2/1 | 1/1 | 3/1 |
|  |  |  |  | Distal | 1/1 | 0/0 | 0/1 | 0/1 | 0/1 | N/A | N/A | N/A | 1/1 | 1/1 | 1/1 | 4/1 |
| FC1072-1 | CMT1B | F | 17 | Proximal | 0/0 | 0/0 | 0/0 | 0/0 | 0/0 | 0/0 | 0/0 | 0/0 | 0/0 | 0/0 | N/A | N/A |
|  |  |  |  | Mid | 0/0 | 0/0 | 0/0 | 0/0 | 0/0 | 0/0 | 0/0 | 0/0 | 0/0 | 1/1 | 1/1 | 1/1 |
|  |  |  |  | Distal | 0/0 | 0/0 | 0/0 | 0/0 | 0/0 | N/A | N/A | N/A | 0/0 | 1/1 | 1/1 | 1/1 |
| FC263-1 | CMT1B | M | 26 | Proximal | 2/2 | 1/1 | 1/1 | 1/1 | 1/1 | 0/0 | 0/0 | 1/1 | 0/0 | 0/1 | N/A | N/A |
|  |  |  |  | Mid | 2/2 | 1/2 | 1/1 | 1/2 | 1/1 | 1/1 | 1/1 | 1/1 | 2/2 | 2/2 | 2/2 | 2/2 |
|  |  |  |  | Distal | 2/2 | 2/2 | 2/2 | 2/2 | 2/2 | N/A | N/A | N/A | 2/2 | 1/1 | 1/1 | 1/1 |
| FC508-1 | CMT1B | F | 21 | Proximal | 1/1 | 1/1 | 1/1 | 1/1 | 1/1 | 1/1 | 1/1 | 1/1 | 1/0 | 1/1 | N/A | N/A |
|  |  |  |  | Mid | 1/1 | 1/1 | 1/1 | 1/1 | 1/1 | 1/1 | 1/1 | 1/1 | 1/1 | 1/1 | 1/1 | 1/1 |
|  |  |  |  | Distal | 1/2 | 1/1 | 1/1 | 1/1 | 1/1 | N/A | N/A | N/A | 1/1 | 1/1 | 1/1 | 1/1 |
| FC533-1 | CMT1B | F | 23 | Proximal | 3/2 | 1/1 | 2/1 | 2/2 | 3/1 | 1/1 | 1/1 | 1/1 | 1/2 | 2/2 | N/A | N/A |
|  |  |  |  | Mid | 2/2 | 1/1 | 2/2 | 2/2 | 2/2 | 1/1 | 1/1 | 1/1 | 1/1 | 1/2 | 1/1 | 1/2 |
|  |  |  |  | Distal | 2/2 | 2/1 | 2/2 | 2/2 | 2/3 | N/A | N/A | N/A | 1/1 | 1/2 | 1/2 | 2/2 |
| FC533-2 | CMT1B | F | 4 | Proximal | 0/0 | 0/0 | 0/0 | 0/0 | 0/0 | 0/0 | 0/0 | 0/0 | 0/0 | 0/0 | N/A | N/A |
|  |  |  |  | Mid | 1/0 | 0/0 | 0/0 | 0/0 | 0/0 | 0/0 | 0/0 | 1/1 | 1/1 | 0/1 | 0/0 | 1/1 |
|  |  |  |  | Distal | 1/1 | 0/0 | 0/0 | 0/0 | 1/1 | N/A | N/A | N/A | 0/0 | 0/0 | 0/0 | 0/0 |
| FC452-1 | CMT1B | F | 11 | Proximal | 1/1 | 0/1 | 0/0 | 1/1 | 1/1 | 0/0 | 0/0 | 1/1 | 0/0 | 1/0 | N/A | N/A |
|  |  |  |  | Mid | 2/1 | 0/0 | 0/0 | 0/0 | 0/0 | 0/0 | 0/0 | 1/0 | 1/1 | 1/1 | 0/0 | 1/1 |
|  |  |  |  | Distal | 1/1 | 0/0 | 0/0 | 0/0 | 0/0 | N/A | N/A | N/A | 1/1 | 1/1 | 1/1 | 1/1 |
|  |  |  | 12 | Proximal | 1/1 | 0/1 | 0/0 | 1/1 | 1/1 | 0/0 | 0/0 | 1/1 | 0/0 | 1/0 | N/A | N/A |
|  |  |  |  | Mid | 2/1 | 1/0 | 1/0 | 1/1 | 0/0 | 0/0 | 0/0 | 1/0 | 1/1 | 1/1 | 0/0 | 1/1 |
|  |  |  |  | Distal | 1/1 | 0/0 | 1/0 | 1/0 | 0/0 | N/A | N/A | N/A | 1/1 | 1/1 | 1/1 | 1/1 |
| FC201-1 | CMT1B | F | 29 | Proximal | 1/1 | 1/1 | 1/1 | 1/1 | 1/1 | 1/0 | 1/0 | 1/1 | 1/0 | 2/1 | N/A | N/A |
|  |  |  |  | Mid | 1/2 | 1/1 | 1/1 | 1/1 | 1/1 | 1/0 | 1/0 | 1/0 | 1/1 | 2/2 | 1/1 | 2/2 |
|  |  |  |  | Distal | 2/2 | 0/1 | 1/1 | 1/1 | 1/1 | N/A | N/A | N/A | 1/1 | 1/1 | 1/2 | 2/2 |
| FC455-1 | CMT1B | F | 3 | Proximal | 0/0 | 0/0 | 0/0 | 0/0 | 0/0 | 0/0 | 0/0 | 1/1 | 0/0 | 0/0 | N/A | N/A |
|  |  |  |  | Mid | 0/0 | 0/0 | 0/0 | 0/0 | 0/0 | 0/0 | 0/0 | 1/1 | 0/0 | 0/0 | 0/0 | 0/0 |
|  |  |  |  | Distal | 1/1 | 0/1 | 1/1 | 1/1 | 0/1 | N/A | N/A | N/A | 1/1 | 1/0 | 0/0 | 1/1 |
|  |  |  | 9 | Proximal | 3/2 | 1/1 | 2/2 | 2/1 | 2/2 | 1/1 | 1/0 | 2/2 | 1/1 | 1/1 | N/A | N/A |
|  |  |  |  | Mid | 3/3 | 1/1 | 1/2 | 1/1 | 2/2 | 1/1 | 1/1 | 2/2 | 3/3 | 2/2 | 1/1 | 1/1 |
|  |  |  |  | Distal | 2/2 | 1/1 | 2/2 | 2/2 | 2/2 | N/A | N/A | N/A | 2/2 | 2/2 | 2/2 | 2/2 |
| FC1027-1 | CMT1B | F | 20 | Proximal | 0/0 | 0/0 | 0/1 | 1/0 | 0/0 | 0/0 | 0/0 | 0/0 | 0/0 | 0/0 | N/A | N/A |
|  |  |  |  | Mid | 1/1 | 1/1 | 1/1 | 1/1 | 1/1 | 1/1 | 1/1 | 1/1 | 1/1 | 1/1 | 1/1 | 1/1 |
|  |  |  |  | Distal | 1/1 | 1/1 | 1/1 | 1/1 | 1/1 | N/A | N/A | N/A | 1/1 | 1/1 | 1/1 | 1/1 |
| FC1159-1 | CMT1B | M | 28 | Proximal | 2/1 | 1/1 | 1/1 | 1/1 | 1/1 | 0/1 | 0/1 | 0/0 | 0/0 | 0/0 | N/A | N/A |
|  |  |  |  | Mid | 1/2 | 1/1 | 1/1 | 1/2 | 1/1 | 1/1 | 1/1 | 1/1 | 1/1 | 2/2 | 1/1 | 2/2 |
|  |  |  |  | Distal | 2/3 | 1/1 | 1/1 | 1/1 | 2/2 | N/A | N/A | N/A | 1/2 | 1/1 | 1/2 | 2/3 |
| FC1159-2 | CMT1B | F | 56 | Proximal | 3/2 | 1/2 | 2/3 | 2/3 | 2/3 | 1/1 | 1/1 | 1/1 | 0/0 | 3/2 | N/A | N/A |
|  |  |  |  | Mid | 3/2 | 3/3 | 3/3 | 4/4 | 3/3 | 2/2 | 2/2 | 3/2 | 1/1 | 4/4 | 3/3 | 1/2 |
|  |  |  |  | Distal | 4/3 | 4/4 | 4/4 | 4/3 | 4/4 | N/A | N/A | N/A | 1/1 | 2/2 | 4/3 | 2/2 |
| FC943-1 | CMTDID | M | 77 | Proximal | 1/1 | 1/1 | 1/1 | 1/1 | 1/1 | 1/1 | 1/1 | 1/1 | 1/1 | 1/1 | N/A | N/A |
|  |  |  |  | Mid | 1/1 | 1/1 | 1/1 | 1/1 | 1/1 | 1/1 | 1/1 | 1/1 | 1/1 | 2/2 | 1/1 | 2/2 |
|  |  |  |  | Distal | 1/1 | 1/1 | 1/1 | 1/1 | 1/1 | N/A | N/A | N/A | 1/1 | 1/1 | 1/1 | 1/2 |
| FC658-1 | CMT2I | M | 60 | Proximal | 0/0 | 0/0 | 0/0 | 0/0 | 0/0 | 0/0 | 0/0 | 1/0 | 0/0 | 1/1 | N/A | N/A |
|  |  |  |  | Mid | 1/1 | 0/0 | 0/0 | 0/0 | 0/0 | 1/0 | 1/0 | 1/1 | 1/1 | 1/1 | 1/1 | 1/1 |
|  |  |  |  | Distal | 1/0 | 0/0 | 0/0 | 0/0 | 0/0 | N/A | N/A | N/A | 0/0 | 1/1 | 1/1 | 1/1 |

Right/left side. The presence of fatty infiltration based on a five-point semiquantitative scale described by Goutallier et al^25^: grade 0, normal; grade 1, some fatty streaks; grade 2, less fat than muscle; grade 3, fatty degeneration of 50%; and grade 4, fatty in ﬁltration of more than 50%. Evaluated muscles at calf level were as follows: proximal calf muscles: tibialis anterior, extensor digitorum longus, peroneus longus, gastrocnemius medial and lateral heads, soleus medial and lateral heads, and tibialis posterior; distal calf muscles: tibialis anterior, extensor digitorum longus, extensor hallucis longus, peroneus longus, soleus medial and lateral heads, tibialis posterior, flexor digitorum longus, and flexor hallucis longus.

Abbreviations: CMT: Charcot-Marie-Tooth disease, F: female, M: male, N/A: not applicable.

**Table S2.** Calf MRI features in Charcot-Marie-Tooth patients with *MPZ* mutations

| Patient | Subtype | Sex | Age at MRI | Level | Anterior compartment | | | Lateral compartment | Superficial posterior compartment | | | | Deep posterior compartment | | |
| --- | --- | --- | --- | --- | --- | --- | --- | --- | --- | --- | --- | --- | --- | --- | --- |
|  |  |  |  |  | Tibialis anterior | Extensor digitorum longus | Extensor hallucis longus | Peroneus longus | Gastrocnemius medial | Gastrocnemius lateral | Soleus medial | Soleus lateral | Tibialis posterior | Flexor digitorum longus | Flexor hallucis longus |
| FC619-1 | CMT1B | M | 3 | Proximal | 0/0 | 0/0 | N/A | 0/0 | 0/0 | 0/0 | 0/0 | 0/0 | 0/0 | N/A | N/A |
|  |  |  |  | Distal | 0/0 | 0/0 | 0/0 | 0/0 | N/A | N/A | 0/0 | 0/0 | 0/0 | 0/0 | 0/0 |
|  |  |  | 7 | Proximal | 0/0 | 0/0 | N/A | 0/0 | 1/1 | 1/1 | 1/1 | 1/1 | 0/0 | N/A | N/A |
|  |  |  |  | Distal | 0/0 | 0/0 | 0/0 | 0/0 | N/A | N/A | 0/0 | 0/0 | 1/1 | 1/1 | 1/1 |
| FC611-1 | CMT1B | F | 3 | Proximal | 0/0 | 1/0 | N/A | 1/1 | 1/1 | 0/1 | 2/2 | 2/2 | 0/0 | N/A | N/A |
|  |  |  |  | Distal | 0/1 | 0/0 | 0/0 | 0/0 | N/A | N/A | 2/1 | 2/1 | 1/0 | 1/0 | 1/0 |
| FC133-4 | CMT1B | M | 15 | Proximal | 0/0 | 0/0 | N/A | 0/0 | 0/0 | 0/0 | 0/0 | 0/0 | 0/0 | N/A | N/A |
|  |  |  |  | Distal | 0/0 | 0/0 | 0/0 | 0/0 | N/A | N/A | 0/0 | 0/0 | 0/0 | 0/0 | 0/0 |
| FC133-10 | CMT1B | M | 19 | Proximal | 0/1 | 1/1 | N/A | 1/1 | 1/1 | 1/1 | 1/1 | 1/1 | 0/1 | N/A | N/A |
|  |  |  |  | Distal | 1/1 | 1/1 | 1/1 | 2/2 | N/A | N/A | 2/1 | 1/1 | 2/2 | 1/0 | 1/0 |
| FC1072-1 | CMT1B | F | 17 | Proximal | 0/0 | 0/0 | N/A | 0/0 | 0/0 | 0/0 | 0/0 | 0/0 | 0/0 | N/A | N/A |
|  |  |  |  | Distal | 0/0 | 0/0 | 0/0 | 0/0 | N/A | N/A | 0/0 | 0/0 | 0/0 | 0/0 | 0/0 |
| FC263-1 | CMT1B | M | 26 | Proximal | 1/1 | 1/1 | N/A | 1/2 | 1/1 | 1/1 | 2/2 | 2/2 | 1/1 | N/A | N/A |
|  |  |  |  | Distal | 2/2 | 1/2 | 1/2 | 2/1 | N/A | N/A | 2/2 | 2/2 | 1/2 | 1/2 | 1/2 |
| FC508-1 | CMT1B | F | 21 | Proximal | 1/1 | 1/1 | N/A | 1/1 | 1/1 | 1/1 | 1/1 | 1/1 | 1/1 | N/A | N/A |
|  |  |  |  | Distal | 1/1 | 1/1 | 1/1 | 1/1 | N/A | N/A | 1/1 | 1/1 | 1/1 | 1/1 | 1/1 |
| FC533-1 | CMT1B | F | 23 | Proximal | 1/1 | 1/2 | N/A | 2/2 | 1/2 | 1/1 | 2/3 | 2/3 | 1/1 | N/A | N/A |
|  |  |  |  | Distal | 1/2 | 1/2 | 1/2 | 3/3 | N/A | N/A | 3/3 | 3/3 | 2/2 | 2/2 | 2/2 |
| FC533-2 | CMT1B | F | 4 | Proximal | 0/0 | 0/0 | N/A | 0/0 | 0/0 | 0/0 | 0/0 | 0/0 | 0/0 | N/A | N/A |
|  |  |  |  | Distal | 0/0 | 0/0 | 0/0 | 0/0 | N/A | N/A | 0/1 | 0/1 | 0/0 | 0/0 | 0/0 |
| FC452-1 | CMT1B | F | 11 | Proximal | 0/0 | 0/0 | N/A | 1/1 | 1/1 | 0/0 | 0/0 | 0/0 | 1/1 | N/A | N/A |
|  |  |  |  | Distal | 3/2 | 0/1 | 0/1 | 2/1 | N/A | N/A | 0/0 | 0/0 | 1/1 | 1/1 | 1/1 |
|  |  |  | 12 | Proximal | 0/0 | 0/0 | N/A | 2/1 | 1/1 | 0/0 | 0/0 | 0/0 | 1/1 | N/A | N/A |
|  |  |  |  | Distal | 3/3 | 1/1 | 1/1 | 2/1 | N/A | N/A | 0/0 | 0/0 | 1/1 | 1/1 | 1/1 |
| FC201-1 | CMT1B | F | 29 | Proximal | 0/1 | 0/1 | N/A | 1/1 | 1/1 | 1/1 | 1/1 | 1/1 | 1/1 | N/A | N/A |
|  |  |  |  | Distal | 0/1 | 1/1 | 1/1 | 2/1 | N/A | N/A | 1/1 | 1/1 | 1/1 | 1/1 | 1/1 |
| FC455-1 | CMT1B | F | 3 | Proximal | 0/0 | 0/0 | N/A | 0/0 | 0/0 | 0/0 | 0/0 | 0/0 | 0/0 | N/A | N/A |
|  |  |  |  | Distal | 0/0 | 0/0 | 0/0 | 0/0 | N/A | N/A | 1/1 | 1/1 | 0/0 | 0/0 | 0/0 |
|  |  |  | 9 | Proximal | 1/1 | 1/1 | N/A | 2/2 | 2/2 | 2/2 | 2/2 | 2/2 | 1/1 | N/A | N/A |
|  |  |  |  | Distal | 1/1 | 1/1 | 1/1 | 2/2 | N/A | N/A | 2/2 | 2/2 | 1/1 | 1/1 | 1/1 |
| FC1027-1 | CMT1B | F | 20 | Proximal | 2/2 | 2/2 | N/A | 2/2 | 3/3 | 2/2 | 2/2 | 2/2 | 2/1 | N/A | N/A |
|  |  |  |  | Distal | 3/3 | 3/3 | 3/3 | 3/3 | N/A | N/A | 3/3 | 4/3 | 3/2 | 3/2 | 3/2 |
| FC1159-1 | CMT1B | M | 28 | Proximal | 4/5 | 2/2 | 3/3 | 3/4 | 3/3 | 2/1 | 2/3 | 2/3 | 1/3 | N/A | N/A |
|  |  |  |  | Distal | 3/3 | 4/4 | 4/4 | 4/4 | N/A | N/A | 3/4 | 3/4 | 1/4 | 2/4 | 2/4 |
| FC1159-2 | CMT1B | F | 56 | Proximal | 4/4 | 4/4 | 4/4 | 4/4 | 4/4 | 4/4 | 2/2 | 2/2 | 2/2 | 2/2 | 2/2 |
|  |  |  |  | Distal | 4/4 | 4/4 | 4/4 | 4/4 | N/A | N/A | 2/1 | 1/1 | 4/4 | 2/2 | 2/2 |
| FC943-1 | CMTDID | M | 77 | Proximal | 1/1 | 1/1 | N/A | 2/2 | 3/3 | 3/2 | 2/2 | 2/2 | 1/1 | N/A | N/A |
|  |  |  |  | Distal | 4/4 | 4/4 | 4/4 | 4/4 | N/A | N/A | 4/4 | 4/3 | 2/2 | 2/2 | 2/2 |
| FC658-1 | CMT2I | M | 60 | Proximal | 1/1 | 1/1 | N/A | 2/1 | 3/3 | 2/2 | 4/4 | 4/4 | 4/4 | N/A | N/A |
|  |  |  |  | Distal | 1/1 | 3/3 | 3/3 | 2/2 | N/A | N/A | 4/4 | 4/4 | 4/4 | 4/4 | 4/4 |

Right/left side. The presence of fatty infiltration based on a five-point semiquantitative scale described by Goutallier et al^25^: grade 0, normal; grade 1, some fatty streaks; grade 2, less fat than muscle; grade 3, fatty degeneration of 50%; and grade 4, fatty in ﬁltration of more than 50%. Evaluated muscles at calf level were as follows: proximal calf muscles: tibialis anterior, extensor digitorum longus, peroneus longus, gastrocnemius medial and lateral heads, soleus medial and lateral heads, and tibialis posterior; distal calf muscles: tibialis anterior, extensor digitorum longus, extensor hallucis longus, peroneus longus, soleus medial and lateral heads, tibialis posterior, flexor digitorum longus, and flexor hallucis longus.

Abbreviations: CMT: Charcot-Marie-Tooth disease, F: female, M: male, N/A: not applicable.
